# Incidence of type 2 diabetes mellitus and prediabetes in Kerala, India: results from a 10-year prospective cohort

**DOI:** 10.1186/s12889-019-6445-6

**Published:** 2019-01-31

**Authors:** Gadadharan Vijayakumar, Sreeja Manghat, Revathi Vijayakumar, Leena Simon, Liss Maria Scaria, Aswathi Vijayakumar, Ganapathy K. Sreehari, V. Raman Kutty, Arun Rachana, Abdul Jaleel

**Affiliations:** 1 Medical Trust Hospital and Diabetes Care Centre, Kulanada, Pathanamthitta (district), Kerala 689503 India; 2 Achutha Menon Centre, Sree Chitra Tirunal Institute for Medical Sciences and Technology, Thiruvananthapuram, India; 3 Christian Medical College, Vellore, Tamil Nadu India; 4 Rajiv Gandhi Centre for Biotechnology, Thycaud post, Poojappura, Thiruvananthapuram, Kerala 695014 India

**Keywords:** Type 2 diabetes, Prediabetes, Asian Indians, Incidence, Cohort

## Abstract

**Background:**

Kerala, the southern state of India, has experienced sudden rise in the prevalence estimates of diabetes. A cohort study on the incidence of type 2 diabetes mellitus (T2DM) in Kerala state thus aptly bridges the lacuna of incidence estimate of T2DM from a population at risk.

**Methods:**

A 10-year prospective cohort study was carried out in two urban wards of central Kerala. The individuals who participated in the baseline survey in 2007 were again invited for a follow-up study in 2017. The data was analyzed using IBM SPSS Statistics for windows (version 21.0). Logistic regression analysis was used to estimate odds ratios and 95% confidence intervals. Findings are based on the 10-year follow-up data from 869 participants from the cohort.

**Results:**

The overall follow-up and response rate of the study was 68.9 and 86.9% respectively. During the follow-up period, 190 people (21.9%) developed T2DM. The incidence rate of T2DM and impaired fasting glucose (IFG) were 24.5 per 1000 person years and 45.01 per 1000 person years respectively. Nearly 60% of participants with baseline IFG were converted to T2DM group in the follow-up period. Age > 45 years, family history of T2DM, BMI ≥ 25 kg/m^2^ and presence of central obesity emerged as important risk factors for incident T2DM.

**Conclusion:**

High incidence of prediabetes over diabetes observed in this study shows an epidemic trend of T2DM in Kerala, India. It requires an immediate public health action.

## Background

Globally, around 450 million people are suffering from diabetes mellitus. The age-standardized global prevalence of diabetes mellitus among adult population has nearly doubled since year 1980, rising from 4.7 to 8.5% [1]. The greatest increase in the prevalence of diabetes mellitus is reported from low and middle-income countries [1, 2]. Asia, being the epicenter for the epidemics of diabetes, is responsible for more than 60% of the global burden of diabetes mellitus [3, 4]. Type 2 diabetes mellitus (T2DM) is the most common form of diabetes. One of the reasons cited for this increasing trend in the prevalence of T2DM in low and middle income countries is Asia paradox [5]. Asia paradox refers to the rapid socio-economic and demographic changes in the Asian population towards that of a developed economy. This can be evident in its economic development, urbanization, and nutritional transition [6]. India, with 69.2 million people with T2DM, is the country with 2^nd^ highest number of people living with diabetes mellitus worldwide next to China [7]. The federal state of Kerala in India is unique in that the health indicators of Kerala are on par with that of developed countries [8–10]. However, T2DM is now highly visible across all sections of society within Kerala and that implies the existence of Asia paradox in Kerala State. Majority of the data on burden of T2DM in India is derived from the prevalence estimates [11]. Though incidence studies could provide more valid estimates of disease trend, such studies on T2DM are limited. A cohort study on incidence of T2DM in Kerala State thus aptly bridges the lacuna of incidence estimate of T2DM from a population at risk.

## Methods

### Setting

India, representing low and middle income country, is constituted by various geographic/administrative federal units called States. In each State there are geographic/administrative subunits such as District, Corporation/Municipality/Panchayat and Wards. Wards are the smallest unit of administration. Kerala State is one among the southern States in India. The study was conducted in two adjacent Wards of Venmony Panchayat of Alappuzha District in Kerala State that are semi-urban.

### Study design and study participants

Study of Life Style Diseases in Central Kerala (SLICK) is an ongoing epidemiological project conducted under the auspices of Medical Trust Hospital and Diabetes Care Centre, Pathanamthitta District, Kerala State, India. Institutional Ethics committee, constituted as per the norms of Indian Council of Medical Research [12], of Medical Trust Hospital and Diabetes Care Centre had approved the study protocol. Study was conducted after obtaining informed written consent from the study participants. The project was initiated in the year 2007, which involved baseline cross-sectional survey and cohort follow-up. The detailed description and findings of the baseline study was published [13]. In brief, the sample frame was constituted based on electoral roll of year 2007: all citizens aged ≥18 years in two adjacent Wards of Venmony panchayat. Of the 1990 adults, 1645 (Male: 624 (37.9%), Female: 1021(62.1%) residents participated in the baseline door-to-door cross- sectional survey (response rate: 82.7%). The baseline assessments included anthropometric measurements (weight, height, waist circumference and hip circumference), assessment of blood pressure and biochemical parameters such as fasting plasma glucose and fasting total cholesterol. The overall crude prevalence of T2DM was 14.6% (241/1645) and Impaired Fasting Glucose (IFG) was 5.1% (84/1645) [13].

**Fig. 1:**
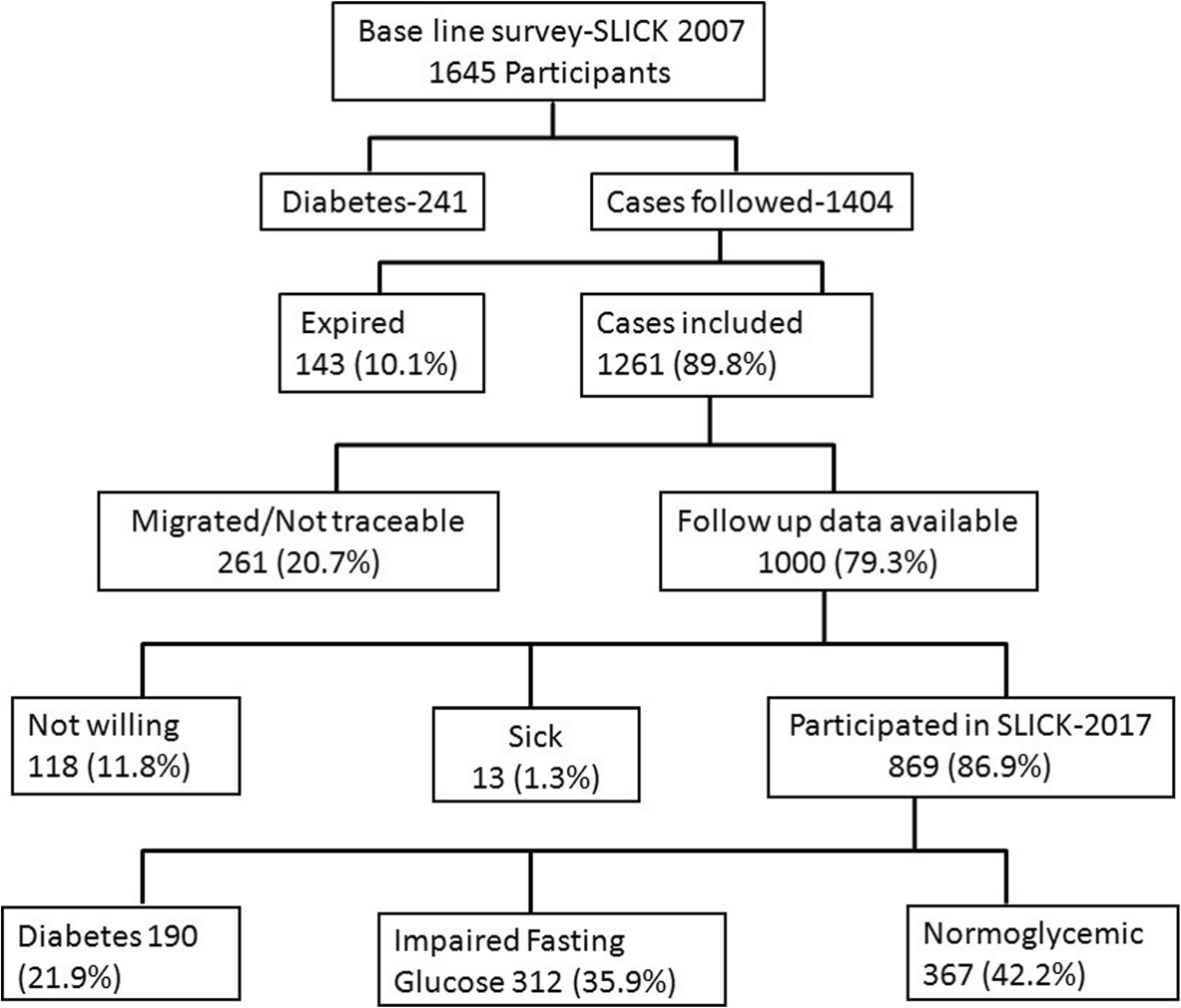
Flow chart on the study participants. The baseline, Study of Life Style Diseases in Central Kerala (SLICK) was conducted in 2007 having 1645 participants. Out of the 1404 participants, who did not have T2DM in 2007, 1000 participants were available for the current study on incidence of T2DM and 869 people were able to participate showing a response rate of 86.9%

The cohort of residents who participated in the baseline survey was re-assessed after a mean period of 10 years. Flow diagram of this cohort is shown in Fig. 1. Accordingly, we intended to follow 1404 participants after excluding 241 participants who were identified as having diabetes in baseline study. In those 1404 eligible participants, 143 (10.2%) participants died before the follow-up study and therefore, 1261 participants became eligible for the follow-up study. While conducting the follow-up study only 1000 participants were accessible as the remaining 261 (18.6%) participants either migrated or were not available. Among the 1000 eligible participants for the follow-up study 118 participants were not willing to participate in the study and 13 participants were sick. The remaining 869 residents (Male: 261 (30.0%), Female: 608 (70.0%)) participated in the study. Thus, the response rate of the follow-up study was 86.9% (869 × 100/1000) and the follow-up rate was 68.9% (869 × 100/1261).

### Study enrolment and data collection

Inclusion criteria for the 10-year follow-up were: those who participated in the baseline survey; those who had normal fasting glucose or impaired fasting glucose in baseline; those who provided informed written consent. Those who were identified as having T2DM during baseline survey were excluded. The data collection period extended from 13th February 2017 to 3rd April 2017. Study enrolment and data collection involved door-to-door visit to eligible participants. In addition to socio-demographic details, data on non-communicable diseases, anthropometry and biochemical parameters were collected. These are described in detail below.

### Assessment of non-communicable disease (NCD) risk factors

NCD risk factors were assessed using a structured interview schedule adapted from World Health Organization STEP instrument for NCD risk factor surveillance [14]. The interview schedule captured behavioral risk factors such as consumption of tobacco and alcohol, physical activity and diet as well as biological risk factors such as personal history of NCDs and family history of NCDs.

### Assessment of anthropometric measurements

By following WHO standard protocol [14], we measured height in meter (using SECA 213 standalone stadiometer), weight in kilogram (using SECA 813 Electronic flat weighing scale) and waist circumference in centimeter (using waist measuring inch tape). Blood pressure (BP) was recorded using Digital Omron apparatus (OMRON-4, Omron Corporation, Kyoto, Japan) as an average of three consecutive readings.

### Assessment of biochemical parameters

Fasting plasma was used to estimate parameters such as Fasting Plasma Glucose (FPG) and Fasting Total Cholesterol (FTC). FPG and FTC were measured respectively by Hexokinase*/*Glucose-6-phosphate dehydrogenase method and cholesterol-peroxidase method using Beckman Coulter AU480 instrument (Beckman Coulter Inc., Ireland).

### Outcome variables

One of the following defines presence of T2DM: Current use of hypoglycemic medication and/or FPG ≥126 mg/dL [15]. Impaired Fasting Glucose (IFG) is defined as FPG in the range 100–125 mg/dL without being on hypoglycemic medication [15]. One of the following defines Hypercholesterolemia: Current use of lipid lowering medication and/or FTC > 200 mg/dL [16]. One of the following satisfies Hypertension: Current use of hypertensive medicine or Systolic blood pressure ≥ 140 mmHg and/or Diastolic blood pressure ≥ 90 mmHg [17]. Central obesity was defined as per Asian Indian cut off for waist circumference (males ≥85 cm and females ≥80 cm) [18]. Body mass index (BMI) (kg/m^2^) was calculated with patient’s weight (kg) divided by height squared (m^2^). BMI was categorized into three groups such as Underweight (BMI < 18.5), Normal (BMI: 18.5–24.9) and Overweight/Obese (BMI ≥ 25) [19].

### Statistical analysis

Data analyses were done using IBM SPSS Statistics for windows v21.0. The descriptive characteristics of participants were described in frequency and percentages. Paired ‘t’ test was used to compare the continuous variables and Pearson chi-square test was used for the categorical variables between baseline year and follow-up year. One way ANOVA was used for comparing present glycemic status of the participants with their baseline characteristics. Person time denominator for assessing incidence rate estimated based on the assumption that T2DM (event) occurred at the mid-point of the interval (5 years). Unadjusted relative risks were estimated from bivariate analysis. Logistic regression models, using all variables in bivariate analysis, were used to estimate the odds ratio with 95% confidence interval. Stepwise forward likelihood ratio method was used for developing the model. Adjusted relative risks were estimated from odds ratio using Zhang-YU method using the equation: RR = OR/ (1 - P_0_) + (P_0_ X OR), where OR refers to Odds ratio derived from logistic regression model and P_0_refers to incidence of outcome of interest (T2DM) in the non-exposed group [20]. We estimated population attributable risk (PAR) for modifiable risk factors using Miettinen’s formula [21]. Population attributable risk, PAR = Pd X (RR-1/RR), where Pd is proportion of cases exposed to a risk factor and RR is adjusted relative risk.

## Results

Results are based on the 10-year follow-up data obtained from the 869 participants of the cohort. Mean age of the participants was 54.50 ± 14.47 years. Table 1 shows demographic and behavioral characteristics of the study participants. Among current users of tobacco, mean ages for initiating smoking and smokeless tobacco are 21.83 ± 10.57 years and 37.52 ± 17 years respectively. The average duration of alcohol consumption among current alcohol users is 22.04 ± 11.5 years.

**Table 1:** Demographic and behavioral characteristics of study participants (*n* = 869) on follow-up survey

Characteristics	Frequency	(%)
Age		
Age Up to 44 years	245	28.2
Age 45 to 59 years	295	33.9
Age 60 and above	329	37.9
Sex		
Male	261	30.0
Female	608	70.0
Education		
Up to 10 years of school education	623	71.7
Greater than 10 years of school education	246	28.3
Diet		
Vegetarian	55	6.3
Non vegetarian	814	93.7
Smoking & Alcohol		
Ever smoker	152	17.5
Current smoker	61	7.0
Ever use of smokeless tobacco	120	13.8
Current use of smokeless tobacco	61	7.0
Ever use of alcohol	105	19.0
Current use of alcohol (within 30 days)	82	9.4
Physical activity		
Low (< 600 MET# minutes/week)	315	36.2
Moderate (600–2999 MET minutes/week)	254	29.2
High (≥3000 MET minutes/week)	300	34.5

Current smokers = Smoked beedi, cigarettes or others within the past 30 days. Current users of smokeless tobacco = Used smokeless tobacco products such as chewing tobacco and betel leaves, snuff, kaini or others within the past 30 days

*MET* Metabolic Equivalent Task

### Physical and biochemical characteristics of the participants

Significant changes (*p* < 0.001) were observed in BMI, Waist circumference, Systolic blood pressure, Diastolic blood pressure, FPG and FTC from baseline to the 10-year follow-up period (Table 2). Increase in age can be one of the factors for these differences in the physical and biochemical characteristics.

**Table 2:** Comparison of participants’ physical and biochemical characteristics between baseline survey (2007) and follow-up survey (2017)

Variables (*N* = 869)	Baseline (2007) Mean (Standard deviation)	Follow-up (2017) Mean (Standard deviation)	*p*-value
Body Mass Index	23.69 (4.20)	24.74 (4.54)	< 0.0001
Waist circumference	80.55 (10.50)	88.65 (12.59)	< 0.0001
Systolic blood pressure	127.79 (19.24)	141.69 (25.11)	< 0.0001
Diastolic blood pressure	78.48 (10.76)	86.17 (13.11)	< 0.0001
Fasting plasma glucose	77.19 (12.69)	113·40 (40·43)	< 0.0001
Fasting total cholesterol	187.44 (32.98)	213·54 (42·95)	< 0.0001

*p*-value is calculated using paired t test

### Incidence of type 2 diabetes mellitus and impaired fasting glucose

During the 10-year mean follow-up period, 190 people developed T2DM, yielding an incidence rate of 24.5 per 1000 person years (190/7740X1000, 95% confidence interval, 21.2–28.2). The cumulative incidence of T2DM was 21.9% (at 95% confidence intervals, 19.1–24.3). The annual incidence of T2DM was 2.9%. Thirty five participants (60%) who had IFG at baseline (*n* = 58) developed T2DM, yielding an incidence rate of T2DM among participants with IFG, 86.41 per 1000 person year (35/405X1000, 95% confidence interval, 61.1–118.9). Incidence rate of T2DM among participants with baseline normal glycemic status is 21.13 per 1000 person year (155/7335X1000, 95% confidence interval, 18.0–24.6). Regarding IFG, 298 (cumulative incidence 36.7%; 95% confidence interval, 33.4–40.2) participants had IFG, yielding an incidence rate of 45.01 per 1000 person years (298/6620X1000; 95% confidence interval, 45.0–50.0).

### Risk factors for developing type 2 diabetes mellitus

Comparison of baseline age, family history of T2DM, anthropometric measurements and biochemical measurements of those who remained normoglycemic, those who converted to T2DM and those who converted to IFG showed that there were significant differences between the groups for all the parameters (Table 3). With respect to the risk factors; age greater than 45 years, central obesity, overweight/obesity (BMI ≥ 25), family history of T2DM and hypertension showed strong association (*p* < 0.001) with the incidence of T2DM (Table 4). Whereas, parameters such as, sex of the participant, physical activity and hypercholesterolemia did not show any relation with the incidence of T2DM (Table 4).

**Table 3:** Comparison of baseline characteristics of the participants (*n* = 811) of normalglycemic status with their glycemic status at follow-up period

Variables	Remained as normal glycemic^a^ (*n* = 358)	Converted to IFG (*n* = 298)	Converted to DM (*n* = 155)	*p* Value
Age	41.82 ± 14·6	44.9 ± 14.0	47.3 ± 13.5	< 0.001
BMI	22.3 ± 3.8	24.0 ± 3.9	25.9 ± 4.6	< 0.001
Waist circumference	77.4 ± 9.9	81.1 ± 10.4	85.7 ± 9·9	< 0.001
Systolic blood pressure	123.5 ± 16.2	129.90 ± 21.8	132.05 ± 18.7	< 0.001
Diastolic blood pressure	76.5 ± 9.6	79·3 ± 11·2	80.7 ± 10.6	< 0.001
Fasting plasma glucose	73.56 ± 8.9	75.19 ± 10.4	77.9 ± 10.3	< 0.001
Fasting total cholesterol	183.0 ± 30.9	189.5 ± 32.9	191.48 ± 35.9	< 0.008
Family history of diabetes^b^	110 (30.8%)	94 (31.6%)	65 (41.9%)	0.038

^a^comparison group. Statistical analyses is done using One way ANOVA

^b^Analyses is done by chi-square test

**Table 4:** Incidence of diabetes mellitus and its associated risk factors at baseline

Risk factors	Incidence (N)	Percentage (%)	Un adjusted RR (95% CI)	Adjusted RR (95%CI)	*p* value
i) Non-modifiable risk factors					
Age					
18–45 years	86	17.6	1	1	
> 45 years	104	27.3	1.55 (1.20–1.99)	1.76 (1.21–2.56)	0.001
Sex					
Female	130	21.4	1	1	
Male	60	23	0.93 (0.71–1.21)	1.32 (0.90–1.92)	0.599
Family history of diabetes					
Absent	107	18.7	1	1	
Present	83	28.2	1.51 (1.17–1.94)	1.63 (1.15–2.32)	0.001
ii) Modifiable risk factors					
Central obesity					
Absent	62	13.6	1	1	
Present	128	30.9	2.26 (.0.72–2.98)	1.59 (1.03–2.43)	0.001
Obesity					
Absent (BMI < 25.0)	81	14.6	1	1	
Present (BMI ≥25.0)	109	34.7	2.37 (1.84–3.05)	2.28 (1.49–3.47)	0.001
Physical activity					
Physical activity ≥600MET/week	169	21.8	1	1	
Physical activity < 600 MET/week	20	23	1.05 (0.70–1.58)	1.17 (0.66–2.06)	0.796
Hypertension					
Absent	116	19.1	1	1	
Present	74	28.1	1.47 (1.14–1.89)	1.08 (0.73–1.59)	0.003
Hypercholesterolemia					
Absent	111	19.9	1	1	
Present	79	25.5	1·28 (0.99–1.65)	1.04 (0.73–1.4)	0.055

Statistical analyses is done using Chi Square test

*T2DM* type 2 diabetes mellitus, *MET* metabolic equalent task, *BMI* Body mass index

**Fig. 2:**
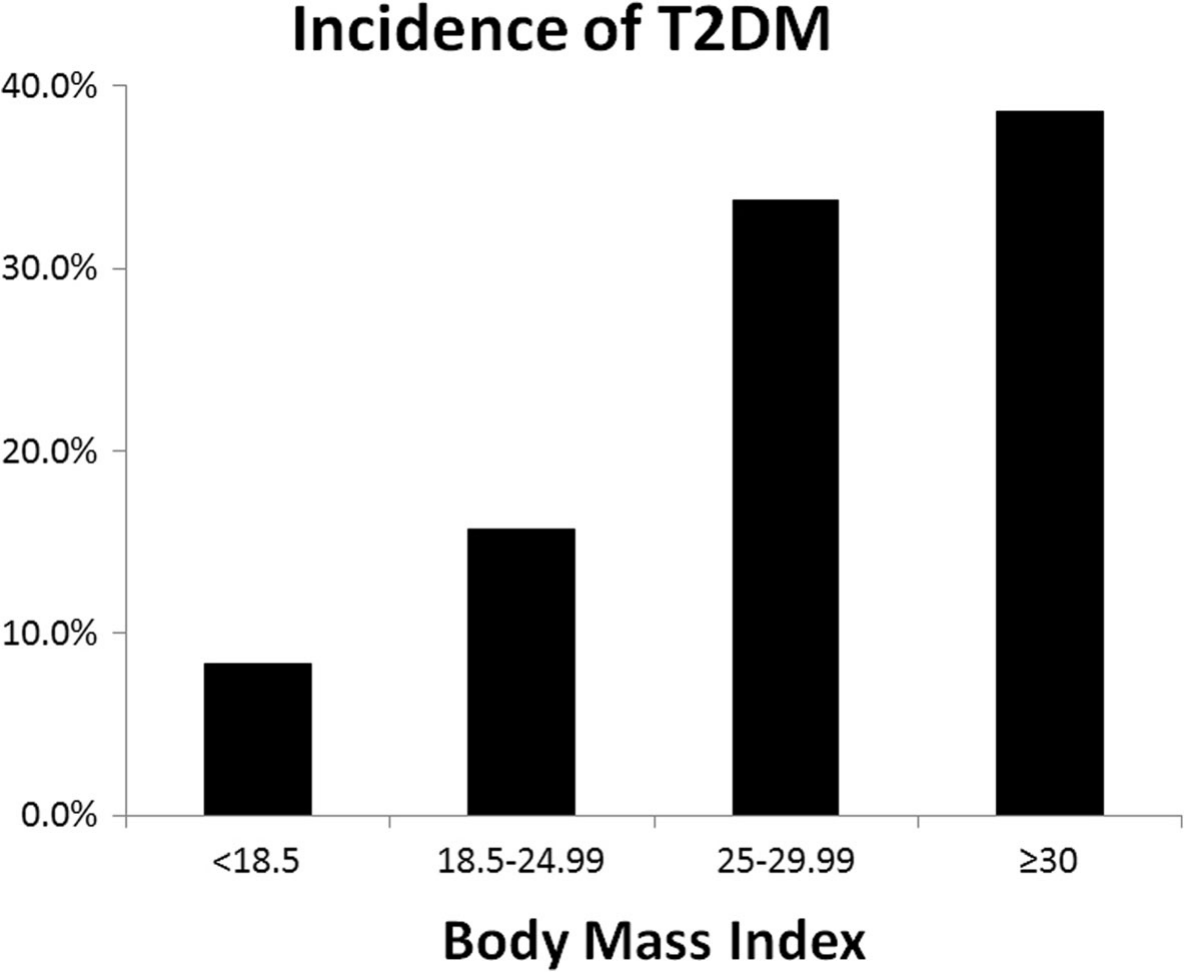
Relationship between baseline BMI and the incidence of T2DM. Study participants were categorized into different BMI groups based on their baseline BMI in 2007 (X-Axis). Y-axis shows the % of people who contracted T2DM in the follow-up period during the current study

Participants with higher baseline BMI had higher incidence of T2DM (Fig. 2). The population attributable risks for overweight/obesity and central obesity were 27.2 and 21.8% respectively (Table 5). Since cholesterol and blood pressure can be intermediary risk factors for obesity, we estimated relative risk and population attributable risk for overweight/obesity adjusted and unadjusted for cholesterol and blood pressure. The relative risk of BMI ≥ 25 adjusted for Cholesterol and Blood pressure was 1·90 (at 95% confidence interval, 1·38–2·50) and the corresponding population attributable risk was 27.2% (PAR = 109/190X (1.90–1/1.90) = 27.2%). Whereas, the relative risk for BMI ≥ 25 unadjusted for Cholesterol and Blood pressure was 1.88 (at 95% confidence interval, 1·38–2·48) and the corresponding population attributable risk was 26.9% (PAR = 109/190 X (1. 88–1/1.88) = 26.9%).

**Table 5:** Results of multivariate analysis: baseline risk factors for incidence of diabetes mellitus

Risk factors	Adjusted RR (95% CI)	PAR (%)
Age group		
18–45 years	1	
> 45 years	1·59 (1·23–2·01)	–
Family H/O DM		
Absent	1	
Present	1·45 (1·36–1·85)	–
BMI ≥ 25		
Absent	1	
Present	1·90 (1·38–2·50)	27.2
Central Obesity		
Absent	1	
Present	1·48 (1·04–2·05)	21.8

Variables adjusted in the model are sex, physical activity, dyslipidemia and hypertension

### Comparison of responders versus non-responders

We observed significant difference in age, sex, BMI, FBS, cholesterol and religion between responders and non-responders (Table 6). It was noted that mean age of non-responders is comparatively less than the responders group. This can be the reason for the significant difference in other baseline variables such as BMI, FBS and Cholesterol level. Less follow-up for younger participants is mainly due to the huge migration of younger participants for job or marriage. The observed differences in baseline characteristics between responders and non-responders will not make much difference in the overall study result as we have a good 68.9% follow-up rate.

**Table 6:** Comparison between the baseline characteristics of responders and non-responders of the follow-up study

Baseline characteristics	Responders 869	Non responders 392	*P*-Value
Age in years	44.3 (14.3)	39.0 (17.7)	< 0.001
BMI	23.7 (4.2)	22.9 (4.1)	0.003
Average sys B.P	127.7 (19.2)	126.0 (17.9)	0.138
FBS	77.2 (12.6)	75.2 (11.0)	0.006
Total Cholesterol	187.4 (32.9)	180.4 (32.9)	< 0.000
Sex			
Female	608 (74.6)	207 (25.4)	< 0.001
Male	261 (58.5)	185 (41.5)	
Family history of DM			
Present	573 (69.8)	248 (30.2)	0.3
Absent	294 (67.1)	144 (32.9)	
Literacy			
Literate	821 (69.4)	362 (30.6)	0.146
Illiterate	48 (61.5)	30 (38.5)	
Family type			
Nuclear	377 (69.7)	164 (30.3)	0.608
Joint	492 (68.3)	228 (31.7)	
Religion			
Hindu	546 (72.1)	248 (30.2)	0.001
Christian	294 (67.1)	144 (32.9)	
Muslim	120 (59.1)	83 (40.9)	

Student’s t-test done for continuous variable and data presented as Mean and standard deviation

Chi-square test was done for categorical variables and data showed in frequency and percentage

## Discussion

Our study describes the incidence of T2DM and IFG during 10 years of follow-up. Our finding that crude incidence of T2DM is 21.9% or 24.5 per 1000 person years re-emphasizes the high burden of T2DM in India. Similar to this finding, high incidence of T2DM was reported in south India by Mohan et al. in 2008 [22], Ghorpade et al. in 2013 [23] and Anjana et al. in 2015 [24]. The incidence of T2DM from those studies were 20.2, 21.5, and 22.2 per 1000 person years respectively. Our cohort attracts special attention in that inspite of being a semi-urban cohort, the incidence is still high in comparison to the two studies from urban south India [22, 24] and the one from rural south India [23]. It is to be noted that high incidence of T2DM is reported from other low and middle income countries too. Latifi et al. in 2016 reported a T2DM incidence rate of 21.9 per 1000 person year from Iran [25]. These observations confirm that the epidemic trends of T2DM is widespread across India as well as across nations.

With the additional information that nearly 60% of participants with baseline IFG converted to T2DM in 10-year follow-up, it is justifiable to conclude that we are facing an epidemic trend of T2DM. Again, this conversion rate (60% or 86.41 per 1000 person years) is much more than the conversion rates from other studies in India. Mohan et al. reported that 40.5% of baseline prediabetes participants converted to T2DM within 8 years of follow-up [22]. Another recent study from South India reported incidence rate of 58.9% or 78.9 per 1000 person-years [24]. Matching these findings, studies from high income country also reported very high incidence of diabetes among participants with baseline IFG [26, 27]. The high incidence rate of prediabetes as compared to T2DM and the high conversion rate of prediabetes to T2DM signal uncontrolled progressive increase in prevalence of T2DM.

Another important finding from our study is the high crude incidence of IFG (36.7%). The high incidence of IFG as compared to incidence of T2DM (crude incidence of 36.7% vs 21.9%) indicates high incidence of prediabetes in the community. In India, a recent multi centric cross- sectional study done in 15 states documented high prevalence of prediabetes as compared to T2DM (24.7% vs. 7.3%) [28]. Similar findings on high incidence of prediabetes were reported from other Asian countries. A study conducted by Latifi et al. in Iran reported the incidence rate of prediabetes as 40.8 per 1000 person years [25]. A study conducted by Vaidya et al. in China also reported high incidence rate of prediabetes (62.6 per 1000 person year) as compared to incidence rate of T2DM, which is 10.0 per 1000 person years [29]. Another 9-year follow-up study from Iran also reported high incidence of prediabetes, 46.1 per 1000 person-years in men and 36.8 per 1000 person-years in women [30]. Thus prediabetes too is at an epidemic trend in our cohort as elsewhere.

Among all the factors studied, obesity emerged as the single-most modifiable risk factor for T2DM. Prevalence of overweight or obesity is quite high (46%) in this cohort. 10% increase in the prevalence of overweight or obesity within 10 year was observed in this cohort. Also, nearly 70% of participants have central obesity based on Asian Indian cutoff. High prevalence of overweight or obesity (25%) was documented in a study conducted on non-communicable risk factors among adults in Kerala in 2009 [31]. In our study, we found that BMI ≥ 25 and central obesity were the significant risk factors for the incidence of T2DM. A person with BMI ≥25 has nearly 1.9 times higher risk than those whose BMI is below 25 (Table 5). The population attributable risk for BMI ≥ 25 is nearly 27% which shows that reduction in overweight or obesity can significantly reduce the incidence of T2DM. Stratified analysis also showed that BMI is independently associated with T2DM as significant risk factor for both men and women. In contrast to our finding, a study done in Iranian population reported BMI as an independent risk factor only in men [32]. Several cohort study as well as cross-sectional study from all around the world reported overweight or obesity and central obesity as important risk factors for T2DM [22, 23, 25, 28, 29, 33–43]. The drastic increase in the prevalence of central obesity (47.6% vs 70.2%) and overweight or obesity (36.2% vs. 46.1%) in this cohort over 10 years amplifies the grave situation. We believe that reduction in obesity or over weight and abdominal obesity can produce substantial reduction in incidence of T2DM. Our study thus emphasizes the importance of addressing obesity in the primary prevention of T2DM. In our study, we measured only total cholesterol among lipid profile. Since we did not measure triglyceride and HDL-cholesterol, burden of metabolic vascular syndrome [44] was not estimated in our cohort.

We observed high crude incidence of T2DM among those aged 60 and above (27.1%). This is consistent with the study done in Canada by Lipscombe et al. in 2007 where they observed high incidence of T2DM over the age group 50 years [45]. Similar association of age as significant risk factor was documented in several studies [25, 28, 35, 36, 42, 46].

In our study, participants with age higher than 45 years has nearly 1.6 times higher risk of having T2DM. The proportion of those aged 60 and above is 37.9% and that could partly explain the high incidence of T2DM in our study population. Age-adjusted incidence of T2DM in our study came to be 14.56 per 1000 person years. But this would not minimize the relevance of our finding since global demographic trend in modern era is towards increasing life expectancy and ageing of population. Hence the burden of T2DM will ever be continually increasing unless preventive measures are adopted.

Another important non-modifiable risk factors observed in this study is family history of T2DM. Nearly 28% of participants with T2DM have a family history of T2DM and this association is significant. This is consistent to several other studies finding that family history of T2DM is an important risk factor for T2DM [25, 27, 28, 34, 45]. High incidence of T2DM in the present cohort ultimately gets reflected as high rates of family history of T2DM in successive generations, thereby spiraling the T2DM burden.

In the current study we didn’t find genderwise difference in the incidence of T2DM which is in contrast to some studies in Asia that reported high incidence of T2DM among males [23, 25, 29]. Whereas, similar to our study, absence of genderwise difference in the incidence of diabetes was noted in a study done in Iran [32]. Over 10 years, prevalence of hypertension (30.3% vs 59.8%) and hypercholesterolemia (35.7% vs. 70.2%) have doubled. Though we had observed high incidence of T2DM among those with hypertension and hypercholesterolemia, the association is not significant.

The high rate of conversion from IFG at baseline into T2DM at 10-year follow-up identifies people with IFG as the target population for preventive measures. From our study, it is also evident that obese individuals are the single most important target population while envisaging preventive strategies against incident T2DM. Moreover, in resource deficit countries, interventions targeting at-risk population are considered resource efficient. Hence, for states/countries with demographics matched to Kerala, public health preventive interventions targeting people with IFG and/or obesity will be the most effective and resource efficient step in preventing T2DM.

Among Indian states, the state of Kerala is known for its demographic indicators and health indicators progressing towards those of developed countries [10]. Thus the state of Kerala represents a transitional zone between developing countries and developed countries. It is likely that any state/country in transition from developing economy to developed economy might face similar trends in non-communicable diseases. This provides an opportunity for such states/countries to anticipate health outcomes and thereby take precautionary measures. A major strength of our study is the good follow-up rate and response rate. We could follow 68.9% of participants from the baseline survey, which is much higher than any other Indian studies.

## Conclusion

The current prospective study estimates that the cumulative incidence of T2DM in Kerala is 21.9% and the incidence of prediabetes is 36.7%. Nearly 60% of the participants who had impaired plasma glucose at baseline converted to T2DM at present, shows an epidemic trend for T2DM in Kerala. Overweight/obesity and central obesity emerged as the most important modifiable risk factors for developing T2DM in Kerala. The findings of this study provide conclusive evidence that incidence of T2DM and prediabetes is increasing rapidly in developing countries.
